# *Epimedium koreanum* Nakai Water Extract Exhibits Antiviral Activity against Porcine Epidermic Diarrhea Virus *In Vitro* and *In Vivo*

**DOI:** 10.1155/2012/985151

**Published:** 2012-11-29

**Authors:** Won-Kyung Cho, Hyunil Kim, Yu Jeong Choi, Nam-Hui Yim, Hye Jin Yang, Jin Yeul Ma

**Affiliations:** ^1^Korean Medicine (KM)-Based Herbal Drug Research Group, Korea Institute of Oriental Medicine, Yuseong-gu, Daejeon 305-811, Republic of Korea; ^2^Optifarm Solution, 48 Songnam-ri, Seonggeo-eup, Seobuk-gu, Cheonan-si 331-834, Chungcheongnam-do, Republic of Korea

## Abstract

Porcine epidemic diarrhea virus (PEDV) causes diarrhea of pigs age-independently and death of young piglets, resulting in economic loss of porcine industry. We have screened 333 natural oriental herbal medicines to search for new antiviral candidates against PEDV. We found that two herbal extracts, KIOM 198 and KIOM 124, contain significant anti-PED viral effect. KIOM 198 and KIOM 124 were identified as *Epimedium koreanum* Nakai and *Lonicera japonica* Thunberg, respectively. The further plaque and CPE inhibition assay *in vitro* showed that KIOM 198 has much stronger antiviral activity than KIOM 124. Additionally, KIOM 198 exhibited a similar extent of antiviral effect against other subtypes of Corona virus such as sm98 and TGE viruses. Cytotoxicity results showed that KIOM 198 is nontoxic on the cells and suggest that it can be delivered safely for therapy. Furthermore, when we orally administered KIOM 198 to piglets and then infected them with PEDV, the piglets did not show any disease symptoms like diarrhea and biopsy results showed clean intestine, whereas control pigs without KIOM 198 treatment exhibited PED-related severe symptoms. These results imply that KIOM 198 contains strong antiviral activity and has a potential to be developed as an antiviral phytomedicine to treat PEDV-related diseases in pigs.

## 1. Introduction

Porcine epidemic diarrhea virus (PEDV) is the causative agent of porcine epidemic diarrhea, dehydration, vomiting, and high mortality in the piglets [1, 2]. PEDV is known for the family of Coronaviridae containing enveloped, single-stranded RNA [3–5]. Most of newborn piglets infected by PEDV would die and pigs of all ages are also affected and exhibit severe symptom like massive diarrhea and dehydration, resulting in serious damage in the swine industry [6–8]. Until now, there are no effective treatments or vaccines developed to prevent economic loss by PEDV. 

Traditionally, natural oriental herbal medicines have been used for relieving and curing many kinds of symptoms arisen from viral infection including cold, flu, and other virus-related diseases [9–14]. A lot of studies demonstrated that herbal extracts from oriental medicinal plants exert significant antiviral effects on infectious viruses including Influenza virus [15, 16], Coronavirus [17], Human immunodeficiency virus (HIV) [18], Hepatitis B [19] or C [20] virus (HBV or HCV), Herpes simplex virus (HSV) [21], Poliovirus [22], Human adenovirus type 1 [23], and Dengue virus type 2 [24]. Nevertheless, herbal extract with antiviral effect on PED virus has not been reported. 

Several compounds including Mizoribine, Deoxynojirimycin, and Ribavirin are available commercially as antiviral drugs. A lot of reports demonstrated that they have the inhibitory effect on various viruses, including cytomegalovirus (CMV) [25], HIV [26], HCV [27], Respiratory Syncytial Virus (RSV) [28], HSV [29], Influenza B virus [30], and ovine viral diarrhea virus (BVDV) [31]. Especially, both Mizoribine and Ribavirin were known to contain the inhibitory activity on severe acute respiratory syndrome (SARS-) associated coronavirus [32]. The antiviral effect of Ribavirin on PEDV was weakly observed *in vitro *[33]. There is no information on the effect of Mizoribine and Deoxynojirimycin on PEDV.

Traditionally, *Epimedium Koreanum *Nakai has been used to treat aphrodisiac, hypotensives, and neurasthenia. *Epimedium Koreanum *Nakai contains a lot of flavonoids including Icariin, Icariside II, Epimedin, Epimedosides, Hyperoside, Qercetin, and Chlorogenic acid. Recent report showed icariin in *Epimedium Koreanum *Nakaistimulated angiogenesis [34]. Other researcher reported that flavonoids and icariin of *Epimedium Koreanum *Nakai improved the development of osteroblast [35]. Also, Icariside II was found to induce apoptosis in human prostate cancer cells [36]. But, the antiviral effect of *Epimedium Koreanum *Nakai was not reported until now. 

In the present study, we first demonstrate the water extract of *Epimedium Koreanum *Nakai inhibits PED viral production *in vitro* and *in vivo* and has a potential to be develop as a phytomedicine for treatment of diseases arisen from PED viral infection in pigs. 

## 2. Materials and Methods

### 2.1. Cells and Viruses

Vero cells (African green monkey kidney cell line; ATCC CCR-81) and ST-cells (pig testis cell line; ATCC CRL-1746) were purchased from KCLB, Korean Cell Line Bank (Seoul, Republic of Korea) and maintained in alpha-minimum essential medium (Hyclone, Logan, UT) with 5% fetal bovine serum and 100 U/mL of Penicillin and Streptomycin at 37°C with 5% CO_2_. Two strains of PED, KPEDV-9 and sm98 and other subtype TGE viruses were obtained from National Veterinary Research and Quarantine Service in Korea. 

### 2.2. Herbal Extract Preparation

The Korean traditional herbal medicines including 333 single medicinal herbal extract were obtained from Yeongcheon Oriental Herbal Market (Yeongcheon, Korea) and verified by Professor Ki Hwan Bae at the College of Pharmacy, Chungnam National University. Fifty grams of each herb were placed in 1000 mL of water and boiled for 3 h at 115°C using medical heating plate (Gyeongseo Extractor Cosmos-600, Incheon, Korea). After boiling until final volume of extract reaches 100 mL, the solution was filtered using standard testing sieves (150 *μ*m) (Retsch, Haan, Germany) and stored at 4°C before use. For further study including cytotoxicity on mouse liver primary cells, KIOM-198 water extract was lyophilized and its final yield was determined as 30 mg/mL. 

### 2.3. Cell Protection from Cytopathic Effect

Vero cells were seeded in 96 wells with complete confluency. Herbal extract containing KIOM 198 or KIOM 124 was 20-fold diluted (1.5 mg/mL) and added to the Vero cells preincubated with viruses for 1 h. The mixture was further incubated for 48 h or 72 h until cytopathic effect (CPE) formation. 

### 2.4. Plaque Assays

Vero cells seeded with complete confluent condition were infected with PED virus at a multiplicity of infection (MOI) of 0.1. KIOM 198 or KIOM 124 extract (1.5 mg/mL) was added to the cells infected with PED virus and incubated for 72 h or 96 h. The supernatants containing virus were harvested and used for determination of virus yield. Plaque assay was used for comparison of virus propagation yield with or without KIOM 198 or KIOM 124 extract. Plaque assay was performed as below. The supernatants harvested were serially diluted up to 10^6^ and added to Vero cells seeded in 6 well (1 × 10^6^ cell/well). After 1 h incubation, media were removed from infected cells and over-layered with 0.5% agar-containing alpha-MEM media, and incubated for 4-5 days at 37°C. Plaques were fixed with 7% formalin, stained with crystal violet, and counted.

### 2.5. Quantitative Real-Time PCR

Virus-containing supernatants were collected from the cells infected with virus (0.01 or 0.1 MOI) with or without KIOM 198 or KIOM 124 at 0, 24 h, 40 h, and 48 h postinfection. Total RNA was isolated using viral Gene-spin Viral DNA/RNA Extraction Kit (European Biotech Network, Belgium) and used for cDNA synthesis using iScript Reverse transcriptase (iNtRON Biotech, Daejeon, Korea) according to manufacturer's instruction. Real-time PCR using Bio-Rad iQ5 (Bio-Rad Laboratories, Inc., Hercules, CA, USA) was performed by subjecting the reaction mixtures to initial denaturation at 94°C for 3 min, followed by 40 cycles of 94°C for 20 sec, 65°C for 20 sec, and 72°C for 30 sec. The primer sequences specific for Nucleocapsid gene of PED virus are used for PCR and as follows: 5′-CGCAAAGACTGAACCCACTAATTT-3′ for forward, 5′-TTGCCTCTGTTGTTACTTGGAGAT-3′ for reverse [37].

### 2.6. Cytotoxicity Assays

To evaluate toxicity of natural herbal extract on Vero cells, lactate dehydrogenase (LDH) assay was performed according to manufacturer's recommendation (Roche, Mannheim, Germany). The level of LDH released into the media is used as a marker of dead cells. Cells seeded at 96 wells (3 × 10^4^ cells/well) were incubated with diluted herbal extracts for 48 h. After incubating with 5 *μ*L of lysis solution for 15 min, 50 *μ*L of reaction mixture was added and more incubated for 5–10 min. Reaction was terminated with addition of 50 *μ*L stop solution and absorbance at 492 nm was measured using spectrophotometer. For checking cytotoxicity of KIOM 198 on mouse liver primary cells, MTT assay was used. MTT (3-(4,5-dimethylthiazol-2-yl)-2,5-diphenyltetrazolium bromide) was purchased from Sigma (St. Louis, MO, USA) and dissolved in a phosphate-buffered saline (PBS) at a concentration of 5 mg/mL. KIOM 198 extract at different concentrations was added to the cells and incubated for 48 h. And then MTT solutions were added to each well and the cells were incubated for another 4 h. The formazan melted in dimethyl sulfoxide (DMSO) was determined with absorbance at 570 nm.

### 2.7. Establishment of Pig Disease Model and Treatment with Herbal Extract

The procedures used in this study were conducted in accordance with the Guidelines for Animal Experimentation of the Institutional Animal Care and Use Committee (IACUC, KFDA, 2004). Two germ-free piglets per group were dieted with or without 198 for 4 days and were orally infected with 10 LD_50_ PED viruses. KIOM 198 extract was added up to 0.6% of diet. After 24 h and 48 h of virus inoculation and at necropsy, the feces were collected for determination of viral yield. After 24 h, the feces of control or KIOM 198-dieted piglets were visually observed. Necropsy was conducted to check the intestine of piglet infected with virus in the presence and absence of KIOM 198.

### 2.8. HPLC Analysis

The standard compounds of quercetin and icariin were purchased from Sigma Co. (USA) and Korea Food and Drug Administration (KFDA), respectively. Purity (%) of all standard compounds was above 98.0%. The powder of *Epimedium Koreanum *Nakai was obtained from Korea Institute of Oriental Medicine (KIOM). HPLC-grade Acetonitrile was purchased from J. T. Baker (USA). Analytical-grade Trifluoroacetic acid (TFA) was obtained from Sigma. Co. (USA). The distilled water was filtered through a 0.45 *μ*m membrane filter (ADVANTEC, Japan) before analysis. The standard solution of quercetin and icariin was prepared by dissolving 2 mg of each compound in methanol at the concentration to 200 ppm. The powdered *Epimedium Koreanum *Nakai 50 mg was dissolved at the concentration of 50 mg/mL in methanol. Then, the sample solution was filtered through a 0.45 *μ*m PVDF membrane filter before analysis. The experiments were performed with Water HPLC system equipped with a 2695 pump and 996 photodiode array detector (USA). The output signal of the detector was recorded using Waters Empower 1.0 software system. The chromatographic columns used in this experiment are commercially available; one was obtained from RS-tech (Optimapak C_18_, 4.6 × 250 mm, 5 *μ*m, Daejeon, Korea). The column oven temperature was kept at 40°C. The injection volume was 10 *μ*L and the flow rate of the mobile phase was 1.0 mL/min. The wavelength of the UV detector was set at 270 nm. The mobile phase composed of water containing 0.1% trifluoroacetic acid (A) and acetonitrile (B). The run time was 70 min and the mobile phase program was the gradient elution as follows: 10% B (0–5 min), 10–40% B (5–60 min), and 40% (60–70 min) (Table 1).

## 3. Results

### 3.1. Screening of Natural Herbal Extract with Antiviral Activity

To search for novel herbal extract containing antiviral activity against PED virus, we screened 333 different oriental herbal medicines. Vero cells were infected with 1 MOI of PED for 1 h, and each herbal extract was 20-fold diluted and then added to the mixture. At 72 h or 96 h post-incubation, each supernatant having virus was collected and total viral yields were measured as plaque forming unit (PFU) using qRT-PCR [37]. Table 1 represents 27 samples containing antiviral activity, selected from the screening. Especially, among 27 candidates, KIOM 124 and KIOM 198 showed significant antiviral activity. KIOM 124 and KIOM 198 were identified as *Lonicera japonica* Thunberg and *Epimedium Koreanum *Nakai, respectively. 

### 3.2. Both KIOM 124 and KIOM 198 Inhibited Cytopathic Effect by PED Viral Replication in Vero Cells

**Figure 1:**
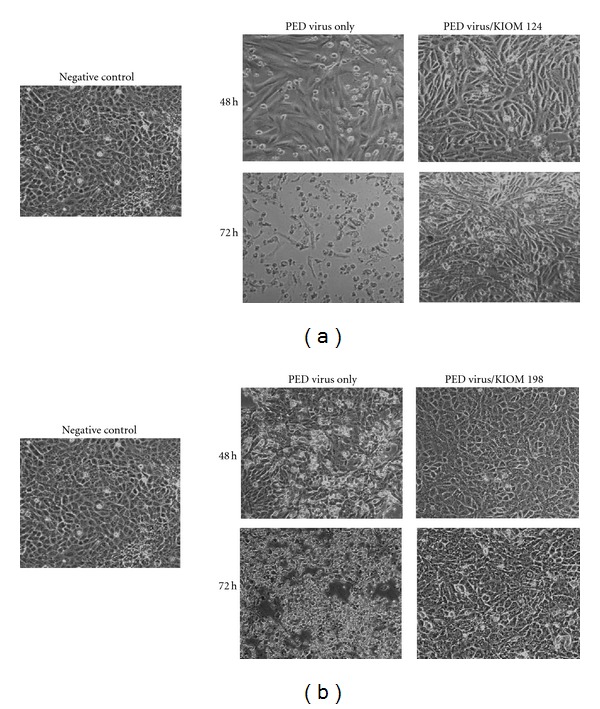
The antiviral activities of KIOM 124 and KIOM 198 on PED virus. The Vero cells were infected with 0.1 MOI of PEDV for 1 h and followed by adding 20-fold diluted herbal extract containing KIOM 124 (a) or KIOM 198 (b), and further incubated for 48 h or 72 h.

KIOM 124 and KIOM 198 were more investigated to confirm the antiviral activity. Using Vero cells infected with virus at an MOI of 0.1, KIOM 124 and 198 further added and incubated for 48 h or 72 h. The control cells infected by virus exhibited partial CPE at 48 h postinfection and full CPE at 72 h postinfection (Figure 1(a), middle panel) but cells containing KIOM 124 did not show CPE effect until 72 h of postinfection (Figure 1(a), right panel).  Figure 1(b) shows KIOM 198 also strongly inhibits cell lyisis by viral replication at 48 h and 72 h postinfection. These results support that KIOM 124 and 198 exert strong inhibitory effect on PED viral replication. 

### 3.3. KIOM 198 Showed Stronger Inhibitory Effect than KIOM 124 on PED Viral Production from 18 h of Postinfection

**Figure 2:**
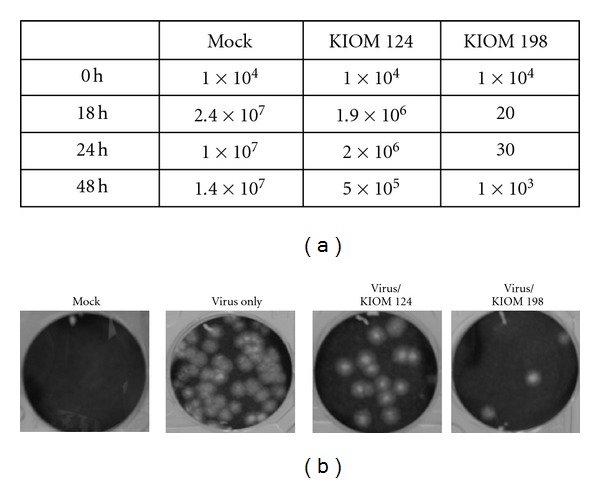
Effect of KIOM 124 and KIOM 198 on viral propagation. Cells infected with 0.1 MOI of virus were further incubated with 20-fold diluted KIOM 124 or KIOM 198 herbal extract. At 18 h, 24 h, and 48 h postinfection, each supernatant was collected and serially diluted for titration. Total viral titers were determined as a plaque forming unit (PFU). (a) Time-course kinetics of virus replicated in the presence or absence of KIOM 124 or KIOM 198 herbal extract. (b) The cells and plaques stained with crystal violet in the presence of KIOM 124 or KIOM 198.

Next, we evaluated the antiviral activities of KIOM 198 and KIOM 124 using plaque assay. The cells preinfected with virus at an MOI of 0.1 were treated with KIOM 124 or KIOM 198 and incubated for 18 h and 24 h. From the supernatant harvested at each time-point, the viral titer was determined by plaque assay.  Figure 2(a) shows when infected with 0.1 MOI of virus, in control cells without KIOM extracts, viral production was steeply increased at 18 h postinfection. The viral replication yield in the cells treated with KIOM 124 was 10 times lower than control. Interestingly, KIOM 198 strongly repressed viral replication at 18 h and 24 h postinfection. Although antiviral effect of KIOM 198 was weaken and viral replication was bounced at 48 h postinfection, it still lowered viral production than control or KIOM 124.  Figure 2(b) is the representative plaque assay results. The plaques formation was repressed 10–40 fold by KIOM 124 and 10^4^ fold by KIOM 198. These findings strengthen both KIOM 124 and 198 have antiviral activity on PED viral replication and KIOM 198 contains much stronger antiviral activity. 

### 3.4. KIOM 198 Inhibited the Propagation of Other Subtype Viruses, SM98 and TGE Viruses

**Figure 3:**
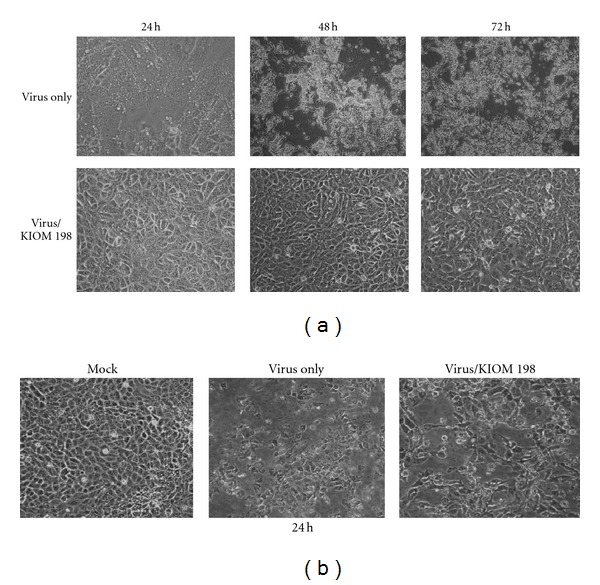
Antiviral effect of KIOM 198 on corona virus other subtype sm98 virus and TGE virus. (a) The Vero cells were infected with sm98 virus at an MOI of 0.01 for 1 h and added with 20-fold diluted KIOM 198 extract, and further incubated for 24 h, 48 h, and 72 h. (b) TGE virus at an MOI of 0.01 was used to infect ST-cells and incubated with KIOM 198 for 24 h.

Since KIOM 198 contains antiviral activity on KPEDV, it is of interest to investigate whether KIOM 198 could inhibit the activity of other subtype of coronavirus. To examine broad inhibitory activity on other corona virus by KIOM 198, other subtype sm98 and TGE viruses were used for antiviral activity test. When cells were infected with sm98 virus, CPE was observed from 24 h postinfection. At 48 h or 72 h postinfection, most cells were lysed by viral propagation (Figure 3(a), top panel). On the contrary, when we added KIOM 198 to the cells, CPE by viral propagation was completely blocked (Figure 3(a), bottom panel). We also tested whether KIOM 198 could protect the cells from CPE by TGE, other subtype of coronavirus. As presented in Figure 3(b), at 24 h postinfection, KIOM 198 inhibited a little CPE by TGE viral propagation. 

### 3.5. Cytotoxicity Results Suggest KIOM 198 Has a Potential to Be Developed for a Safe Antiviral Drug

**Figure 4:**
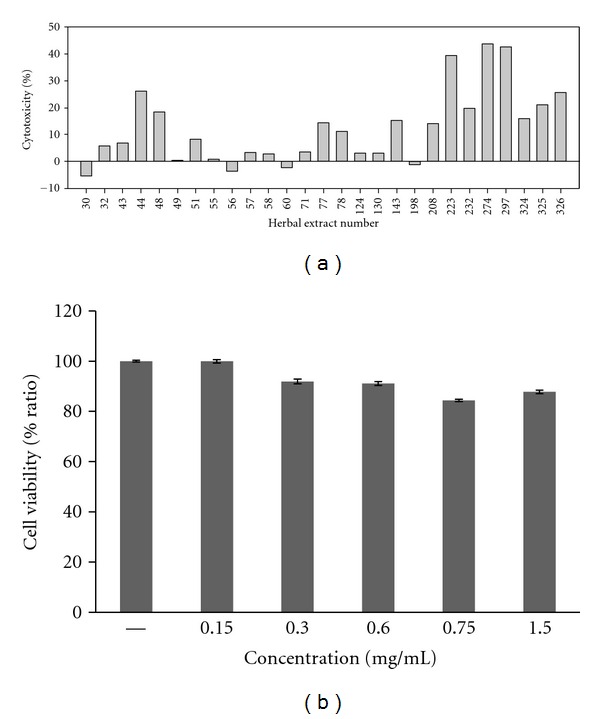
(a) Cytotoxicity of herbal extract containing antiviral activity in Vero cells. The 27 different herbal extracts were evaluated to examine toxicity on the cells. (b) Cytotoxicity of KIOM 198 on mouse primary liver cells.

The antiviral activity test showed that KIOM 198 has a remarkable ability to inhibit coronavirus propagation. Next, we investigated the toxicity of KIOM 198 using lactate dehydrogenase (LDH) assay in Vero cells and MTT assay in mouse primary liver cells. Vero cells were lysed, mixed with 20 fold-diluted herbal extract, and the activity was measured using substrate.  Figure 4(a) represents the cytotoxicity result of 27 antiviral herbal extracts in Vero cells. The extent of cytotoxicity of KIOM 198 is close to nothing, compared to other herbal extracts. And, when we checked the cytotoxicity of KIOM 198 on mouse liver primary cells, in the presence of 1.5 mg/mL, cell viability was more than 90% (Figure 4(b)). These data suggest KIOM 198 could be a safe and nontoxic drug when it is used for antiviral therapy. 

### 3.6. KIOM 198 Repressed PED Viral Replication and Relieved Disease Progress in PED Virus-Infected Pigs

**Figure 5:**
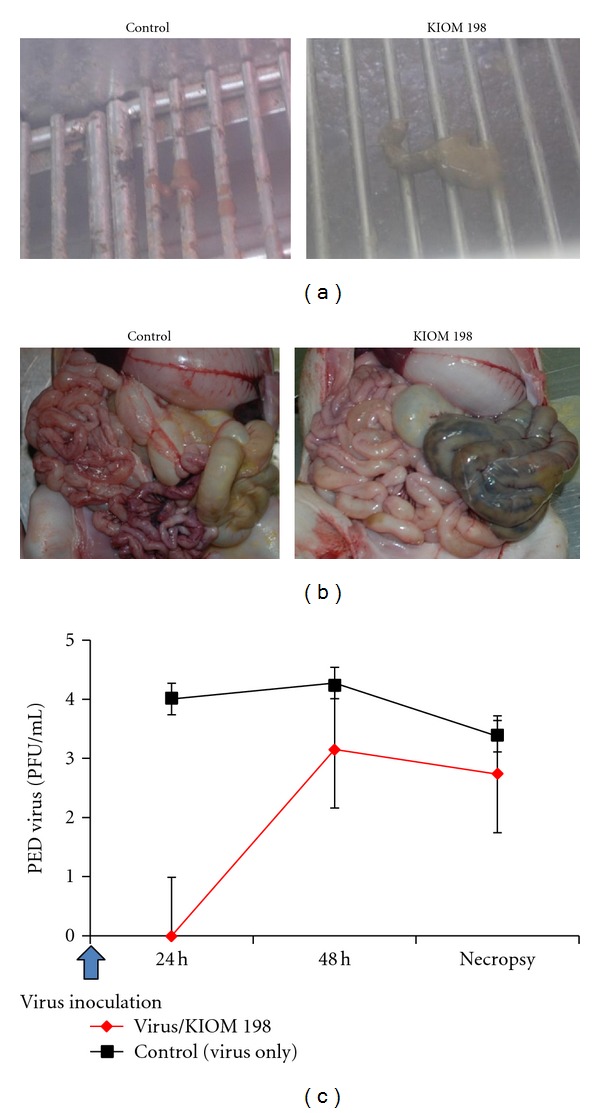
Effect of KIOM 198 on pigs infected by PED virus. The germ-free piglets were orally injected PED virus after 4 days diet with or without KIOM 198. KIOM 198 extract was added up to 0.6% of diet. After 24 h, the feces of control or KIOM 198-dieted piglets were observed (a). Necropsy was performed to check the intestine of piglet infected with virus in the presence and absence of KIOM 198 (b). After 24 h and 48 h of virus inoculation and at necropsy, the feces were collected to determine viral propagation yield (c).

To prove the inhibitory activity of KIOM 198 against PED virus, we established disease model in the piglet. Piglets were infected with 10 LD_50_ PED viruses after 4 days feeding with milk and KIOM 198. At 24 h postinfection, the piglets fed with KIOM 198 had normal feces, whereas control piglets had diarrhea (Figure 5(a)). Furthermore, in the presence of KIOM 198, the intestine of piglet was free of disease symptom (Figure 5(b), right panel) compared to intestine of control pigs, which was thinned and filled with diarrheal material (Figure 5(b), left panel). Furthermore, we compared viral replications in the piglets, at 24 h, 48 h postinfection, and on necropsy. As shown in Figure 5(c), no virus was detected in the feces of piglet dieted with KIOM 198 at 24 h postinfection. From 48 h of postinfection, viral number in the KIOM 198-dieted piglets was increased, but it was still 10-fold lower than control group. Taken together these results, KIOM 198 has strong inhibitory effect on PED viral growth in the pigs.

### 3.7. HPLC Analysis

**Figure 6:**
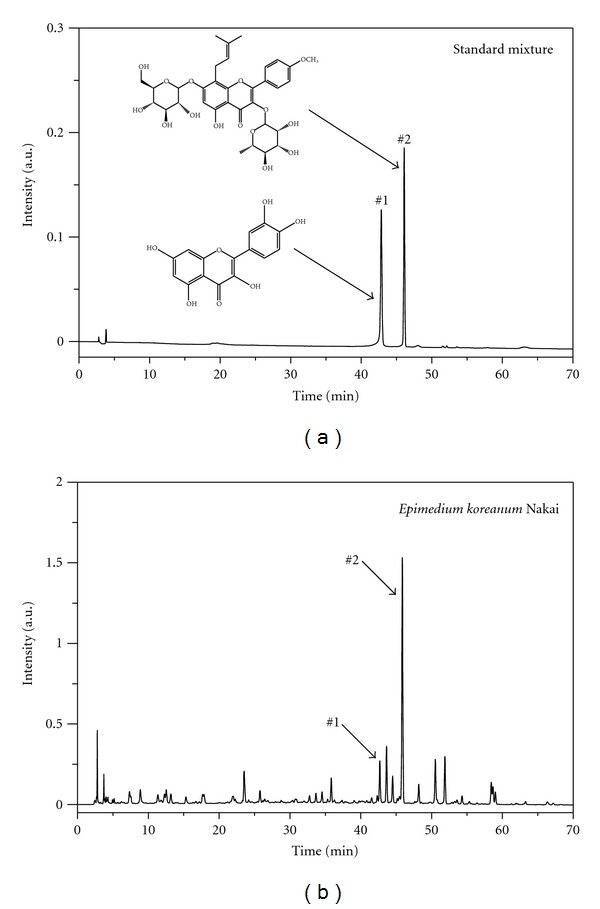
Analysis of the component profile of KIOM 198 by RP-HPLC-DAD (run time: 70 min, mobile phase A: water 99.9% + TFA 0.1%, B: Acetonitrile 100%; 10% B (0–5 min), 10–40% B (5–60 min), and 40% (60–70 min), injection: 10 *μ*L, UV wavelength: 270 nm. Several peaks including quercetin (1) and icariin (2) were detected in KIOM 198.

The main component profile of KIOM 198 was analyzed using HPLC. As shown in Figure 6, among marker compounds of *Epimedium Koreanum *Nakai, quercetin and icariin were representatively identified at 270 nm based on comparison to the standard compounds. Several unidentified peaks were detected. 

## 4. Discussion

In this study, we have represented the antiviral activity of KIOM 198, water extract of *Epimedium Koreanum *Nakai, on PED virus. PEDV causes severe damage to pig-industry. It is inevitable to develop a novel, strong viral inhibitor to prevent economical loss from PED viral infection. Although some inhibitors were screened and tested for antiviral effect on PED virus, new effective antiviral remedy without toxicity should be developed. 

KIOM 198 exhibited antiviral activity on not only PED virus but other corona virus like sm98 and TGE virus. We examined whether antiviral agents available commercially, such as Deoxynojirimycin, Mizoribine, and Ribavirin, could inhibit PED viral growth. Each compound at concentration of 100 *μ*g/mL was treated to the Vero cells infected with PED virus and compared their antiviral activity with KIOM 198. These agents except for KIOM 198 did not significantly inhibit PED viral growth in this study (data not shown). These results mean KIOM 198 contains specific, remarkable antiviral activity on PED virus, which is not inhibited by other well-known antiviral drug. Antiviral activity of KIOM 198 was confirmed *in vivo* disease model. Piglets infected with PED virus expressed disease symptoms like diarrhea, but piglet oral-administered with KIOM 198 had normal feces and no virus was detected in the early time and 10-fold lower viruses were found in the feces later. This result suggests the antiviral effect of KIOM 198 on the virus is very effective in the early stage. 

Importantly, the cytotoxicity of antiviral reagents should be considered prior to use. Traditional oriental herbal medicines have been used for human being for a long time and any severe side-effect after dose did not reported. KIOM 198 showed the least toxicity among 27 antiviral candidates extracted from herbal medicines and also did not have any significant toxicity on normal primary cells. 

We analyzed KIOM 198 using HPLC and detected several peaks including two known compounds, icariin and quercetin. Recent reports demonstrated that Quercetin 7-rhamnoside reduces PED viral replication [33, 38]. They also showed quercetin, apigenin, luteolin, and catechin contained moderate anti-PEDV activity, and ribavirin, coumarin, and tannic acid exhibited weak efficacy on PEDV. Based on these reports, further studies are needed to examine whether active compounds including Quercetin 7-rhamnoside are present in KIOM 198 or which new compounds in KIOM 198 are responsible for anti-PED viral effect. 

Finally, we have demonstrated that KIOM 198, water extract of *Epimedium Koreanum *Nakai, exerts a potent antiviral activity on PED virus *in vitro* and *in vivo* animal model. Despite the fact that the underlying mechanism of KIOM 198 action in details should be addressed, we assume KIOM 198 exerts strong antiviral effect through modulating immune response such as macrophage and lymphocyte stimulation.

## Figures and Tables

**Table 1:** Screening of herbal extract containing antiviral activity against PED virus. 333 herbal extracts were evaluated for the inhibitory effect on PED viral production and 27 herbal extracts having anti-PED viral activity were selected. Antiviral activity was examined by plaque assay and determined as a PFU (plaque forming unit). KIOM 124 and KIOM 198 exhibited strong inhibitory effect on PED viral propagation.

Extract number	PEDV-PFU	PEDV log PFU	Fold inhibition
30	713.1	2.853	410
32	7391	3.869	40
43	2488	3.396	118
44	1736	3.24	168
48	2962	3.472	99
49	20570	4.313	14
51	85600	4.932	3
55	1084	3.035	270
56	2174	3.337	135
57	2678	3.428	109
58	1817	3.259	161
60	6042	3.781	48
71	256.8	2.41	1139
77	749.5	2.875	390
78	47.48	1.676	6160
**124**	**<1**	**<0**	**>292500**
130	133.9	2.127	2184
143	179100	5.253	2
**198 **	**<1**	**<0**	**>292500**
208	2563	3.409	114
223	58240	4.765	5
232	2212	3.345	132
274	344.2	2.537	850
297	431900	5.635	1
324	3582	3.554	82
325	734200	5.866	No inhibition
326	165600	5.219	2
Positive control (No treatment)	292500	5.466	
